# In vivo MRS study of long-term effects of traumatic intracranial injection of a culture medium in mice

**DOI:** 10.18699/VJGB-23-74

**Published:** 2023-10

**Authors:** O.B. Shevelev, O.P. Cherkasova, I.A. Razumov, E.L. Zavjalov

**Affiliations:** Institute of Cytology and Genetics of the Siberian Branch of the Russian Academy of Sciences, Novosibirsk, Russia Institute “International Tomografic Center” of the Siberian Branch of the Russian Academy of Sciences, Novosibirsk, Russia; Institute of Laser Physics of the Siberian Branch of the Russian Academy of Sciences, Novosibirsk, Russia Novosibirsk State Technical University, Novosibirsk, Russia; Institute of Cytology and Genetics of the Siberian Branch of the Russian Academy of Sciences, Novosibirsk, Russia Novosibirsk State University, Novosibirsk, Russia; Institute of Cytology and Genetics of the Siberian Branch of the Russian Academy of Sciences, Novosibirsk, Russia

**Keywords:** magnetic resonance spectroscopy, animal model of human glioma, neurometabolites, traumatic brain injury, магнитно-резонансная спектроскопия; ; ;, животная модель глиобластомы человека, нейрометаболиты, черепно-мозговая травма

## Abstract

Orthotopic transplantation of glioblastoma cells in the brain of laboratory mice is a common animal model for studying brain tumors. It was shown that 1H magnetic resonance spectroscopy (MRS) enables monitoring of the tumor’s occurrence and its development during therapy based on the ratio of several metabolites. However, in studying new approaches to the therapy of glioblastoma in the model of orthotopic xenotransplantation of glioma cells into the brain of mice, it is necessary to understand which metabolites are produced by a growing tumor and which are the result of tumor cells injection along the modeling of the pathology. Currently, there are no data on the dynamic metabolic processes in the brain that occur after the introduction of glioblastoma cells into the brain of mice. In addition, there is a lack of data on the delayed effects of invasive brain damage. Therefore, this study investigates the long-term dynamics of the neurometabolic profile, assessed using 1H MRS, after intracranial injection of a culture medium used in orthotopic modeling of glioma in mice. Levels of N-acetylaspartate, N-acetylaspartylglutamic acid, myoinositol, taurine, glutathione, the sum of glycerophosphocholine and phosphocholine, glutamic acid (Glu), glutamine (Gln), and gamma aminobutyric acid (GABA) indicate patterns of neurometabolites in the early stage after intracranial injection similar to brain trauma ones. Most of the metabolites, with the exception of Gln, Glu and GABA, returned to their original values on day 28 after injection. A progressive increase in the Glu/Gln and Glu/GABA ratio up to 28 days after surgery potentially indicates an impaired turnover of these metabolites or increased neurotransmission. Thus, the data indicate that the recovery processes are largely completed on day 28 after the traumatic event in the brain tissue, leaving open the question of the neurotransmitter system impairment. Consequently, when using animal models of human glioma, researchers should clearly distinguish between which changes in neurometabolites are a response to the injection of cancer cells into the brain, and which processes may indicate the early development of a brain tumor. It is important to keep this in mind when modeling human glioblastoma in mice and monitoring new treatments. In addition, these results may be important in the development of approaches for non-invasive diagnostics of traumatic brain injury as well as recovery and rehabilitation processes of patients after certain brain surgeries.

## Abstract (ru)

Ортотопическая ксенотрансплантация клеток глиобластомы в головной мозг лабораторных мышей – распространенная животная модель для изучения опухолей головного мозга. Показано, что 1Н магнитно-резонансная спектроскопия (МРС) позволяет отслеживать возникновение опухоли и ее развитие в процессе терапии по соотношению нескольких метаболитов. Однако при изучении новых подходов в терапии глиобластомы на модели ортотопической ксенотрансплантации клеток глиомы в головной мозг мышей необходимо понимать, какие изменения уровней метаболитов являются следствием роста опухоли, а какие – результатом инъекции опухолевых клеток в головной мозг в процессе моделирования патологии. В настоящее время отсутствуют данные о динамике метаболических процессов в головном мозге, возникающих после введения клеток глиобластомы в мозг мышей. Мало также данных об отсроченных последствиях инвазивного повреждения головного мозга. Поэтому в нашей работе исследуется долговременная динамика нейрометаболического профиля, оцененного с примене-нием 1H МРС, после внутричерепной инъекции культуральной среды, используемой при ортотопическом моделировании глиомы у мышей. Уровни N-ацетиласпартата, N-ацетиласпартилглутаминовой кислоты, мио-инозитола, таурина, глутатиона, суммы глицерофосфохолина и фосфохолина, глутаминовой кислоты (Glu), глутамина (Gln) и гамма-аминомасляной кислоты (ГАМК) указывают на паттерны нейрометаболитов на ранней стадии после внутричерепной инъекции, схожие с таковыми при травме головного мозга. Большинство метаболитов, за исключением Gln, Glu и ГАМК, возвращались к исходным значениям на 28-й день после инъекции. Прогрессирующее увеличение соотношения Glu/Gln и Glu/GABA до 28 дней после операции потенциально указывает на нарушение обмена этих метаболитов или усиление нейропередачи. Таким образом, данные свидетельствуют о том, что восстановительные процессы в основном завершаются на 28-й день после травматического события в ткани головного мозга, оставляя открытым вопрос о нарушении нейромедиаторной системы. Соответственно, при использовании животных моделей глиомы человека исследователи должны четко различать, какие изменения нейрометаболи тов являются реакцией на саму инъекцию раковых клеток в головной мозг, а какие процессы могут свидетель ствовать о раннем развитии опухоли головного мозга. Это важно иметь в виду при моделировании глиобластомы человека у мышей и мониторинге новых методов лечения. Кроме того, полученные данные могут быть важны при разработке подходов к неинвазивной диагностике черепно-мозговой травмы, а также при мониторинге процессов восстановления и реабилитации пациентов после некоторых операций на головном мозге.

## Introduction

Glioblastoma (GBM) is the most common and aggressive tumor of the central nervous system (Goodenberger, Jenkins, 2012). Even in the case of aggressive therapy of the brain glioma, such as surgical resection, radiotherapy, and chemotherapy, many types of gliomas almost always have a pessimistic prognosis for the patients’ survival (Tykocki et al., 2018; Ostrom et al., 2019). Despite the efforts of scientists and clinicians to increase the life expectancy of GBM patients, survivors do not easily exceed the 15th month (3–5) and the 5-year survival rate is as low as 5.8 % (Ostrom et al., 2018, 2019; Tan et al., 2020).

Xenograft mice models are widely used for modeling GBM and testing developmental therapeutics (Haddad et al., 2021). Orthotopic transplantation of glioblastoma cells in the brain of laboratory mice is a common animal model for studying brain tumors (Zavjalov et al., 2016; Miyai et al., 2017; Haddad et al., 2021). This model is characterized by rapid glioma growth following injection of tumor cells into the subcortical structures of the brain, resulting in destruction of blood vessels and neurons, compression of individual brain structures, impairment of cognitive function and deep irreversible lesions (Hall et al., 2005; Maas et al., 2008). Currently, the most accessible way to detect and visualize glioma is magnetic resonance imaging (MRI), which is widely used in the clinic. The main advantage of MRI is its non-invasiveness with the ability to image unlabeled cells, while optical methods require stable expression of fluorophore-labeled protein in tumor cells, which can lead to a change in the behavior of tumor cells (Dass, Choong, 2007). Finally, MRI is a reliable and sufficiently accurate method that can be used to repeatedly study the dynamic processes for the same organism.

To visualize tumors, reliable MRI contrast agents are used, which, however, do not always allow a correct diagnosis. Up to 45 % of abnormalities in a patient’s brain detectable with MRI require biopsy and subsequent histological studies. There is another significant drawback of this approach for intracerebral tumors. Contrast does not always lead to an increase in the MRI signal, and although this happens quite rarely – about 4 % of gliomas, the life of the patient is on the line in every such case (Barker et al., 1997; Ginsberg et al., 1998; Sugahara et al., 1998; Castillo et al., 2001; Nelson, Cha, 2003; Atkinson et al., 2008; Stockhammer et al., 2008).

Recently, in vivo studies of the functional state of the brain are increasing due to attempts to assess the metabolic profile using 1H magnetic resonance spectroscopy – MRS (Ratai, Gilberto González, 2016). It was shown that MRS enables monitoring of the tumor’s occurrence and its development during therapy based on the ratio of several metabolites (Hishii et al., 2019; Tiwari et al., 2020). However, at study of new approaches in the therapy of glioblastoma in the model of orthotopic xenotransplantation of glioma cells into the brain of mice, it is necessary to understand which metabolites are produced by a growing tumor and which are the result of tumor cells injection along the modeling of the pathology. Currently, there are no data on the dynamic metabolic processes in the brain that occur after the introduction of glioblastoma cells into the brain of mice. Furthermore, there is a lack of data on the delayed effects of invasive brain damage (Singh et al., 2016; Li Y. et al., 2021). These studies require multiple animal MRI sessions under anesthesia, and so this study investigates the long-term dynamics of the neurometabolic profile by 1H MRS repeated sessions after intracranial injection of a culture medium used at orthotopic modeling of glioma in mice.

## Materials and methods

The study was carried out in the Center for Collective Use “SPF-vivarium” of the Institute of Cytology and Genetics, Siberian Branch of the Russian Academy of Sciences (RFMEFI62119X0023). This study was approved by the Inter-Institutional Commission on Biological Ethics at the Institute of Cytology and Genetics, Siberian Branch of the Russian Academy of Sciences (Permission #78, April 16, 2021). Ten SPF male SCID mice aged 6–7 weeks were used. Their health status was investigated in accordance with the recommendations of the Federation of European Laboratory Animal Science Associations (FELASA working group…, 2014). The animals were kept in single-sex family groups of 2–5 mice in individually ventilated cages using the Optimice system (Animal Care Systems, Centennial, CO, USA). The mice were maintained under controlled conditions: temperature, 22–26 °C; relative humidity, 30–50 %; and 12/12 light/ dark periods with dawn at 02:00. Standart V1534-300 ssniff® diet (ssniff Spezialdiäten GmbH, Soest, Germany) and reverse osmosis water enriched with minerals were provided to the animals ad libitum.

Before the operation, the animal was placed in a chamber with an air flow of 300–350 ml/min and an isoflurane concentration of 1.5 % (Baxter International Inc., Deerfield, IL, USA). After 3–5 min, the animal was transferred to a heated operating table with a surface temperature of 37 °C and placed under an anesthetic mask with 1.5 % isoflurane. The culture medium (DMEM\F-12, Thermo Fisher Scientific, Waltham, MA, USA) used for orthotopic glioma modeling was introduced into the subcortical structures of the brain through a hole in the cranium (Zavjalov et al., 2016). A 3–4 mm skin incision was made on the animal’s head in the caudal-cranial direction in the region of the bregma, and 5 μl of medium was injected through the hole in the skull. Intravital MRS was performed on the anesthetized animals before surgery and on days 7, 14, 21 and 28 after the injection.

Magnetic resonance imaging and MRS protocols. Neurometabolite analyses were performed on a horizontal tomograph with a magnetic field strength of 11.7 Tesla (Biospec 117/16; Bruker, Billerica, MA, USA). Five minutes prior to the analysis, animals were immobilized with gas anesthesia (Isofluran, Baxter International Inc.) using an anesthesia machine (The Univentor 400 Anaesthesia Unit; Univentor Ltd., Zejtun, Malta). The body temperature of the animals was maintained using a water circuit in a tomographic bed-table with a surface temperature of 30 °C. A pneumatic breathing sensor (SA Instruments Inc., Stony Brook, NY, USA) was placed under the lower torso, which made it possible to control the depth of anesthesia.

**Fig. 1.**
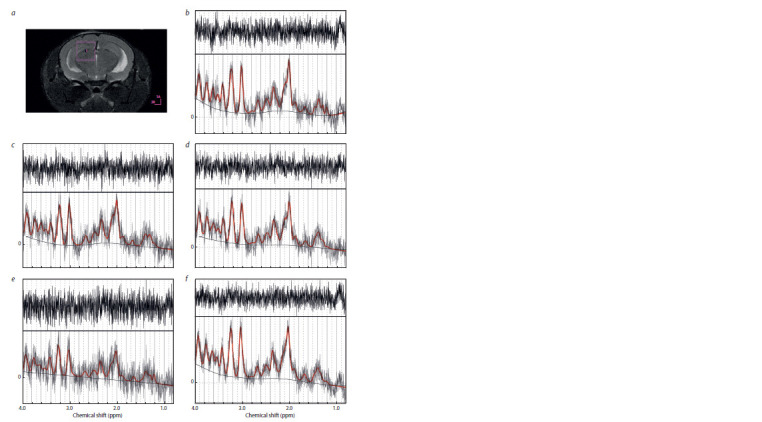
Voxel location (a), characteristic spectra and FIDs (LCModel) before (b) and 7 (c), 14 (d ), 21 (e) or 28 (f ) days after injection.

All proton spectra of the mouse brain were obtained using a 1H volumetric radiofrequency coil (T11440V3). Correct positioning of spectroscopic voxels (2.5 × 2.5 × 2.5 mm3) was performed using the RARE method (rapid acquisition with relaxation enhancement), with the parameters of the pulse sequence set at TE = 11 ms and TR = 2.5 s. T2-weighted high-resolution mouse brain images (slice thickness, 0.5 mm; field of view, 2.0 × 2.0 cm; and matrix size, 256 × 256 points) were obtained. The voxel location is shown in Fig. 1, a. All proton spectra were obtained using spatially localized singlevoxel spectroscopy using the STEAM method (stimulated echo acquisition mode spectroscopy) with pulse sequence parameters of TE = 3 ms, TR = 5 s, and the number of accumulations = 180. Before each spectroscopic measurement, the uniformity of the magnetic field was adjusted within the selected voxel using the FastMap technique (Gruetter, 1993). The water signal in the spectra was suppressed using a variable power pulse and an optimized relaxation sequence delay (VAPOR) (Tkac et al., 1999).

MRS processing. The LCModel software package was used to process the experimental 1H MRS spectra and determine the number of individual metabolites (Provencher, 2001). This software package has a high degree of automation with minimal user intervention, which minimizes biased input data, thus allowing data exchange, acceptance, and reliable comparison with values obtained using different equipment or under different conditions. Metabolites with Cramer-Rao lower bounds, which represent the estimates of the percentage standard deviation of the fit for each metabolite, < 20 % were considered reliable and are presented in this study. MRS processing is described in more detail in (Singh et al., 2016). According to the manual for the LCModel, the following levels were estimated: the sum of N-acetylaspartate and N-acetylaspartylglutamic acid (tNAA), myoinositol (mIno), taurine (Tau), glutathione (GSH), the sum of glycerophosphocholine and phosphocholine (tCho), glutamic acid (Glu), glutamine (Gln), gamma aminobutyric acid (GABA) as a relation to the sum of creatine and creatine phosphate (tCr). Moreover, the ratios of neurotransmitters and their precursors Glu/GABA and Glu/Gln were estimated (see Fig. 1).

Statistical analysis. We used Student’s t-test for dependent samples to process the results. The values of the studied parameters are presented as means ± standard error of the mean.

## Results

In studies using in vivo MRS, data are presented both as concentrations or relative units of individual metabolites, and as the ratio of individual metabolites to tCr, using it as an internal control. Since in our work the level of tCr in animals did not differ from the base level at all the studied time points ( p > 0.19), it would be appropriate in the future to present the data as the ratio of individual metabolites to tCr.

**Fig. 2.**
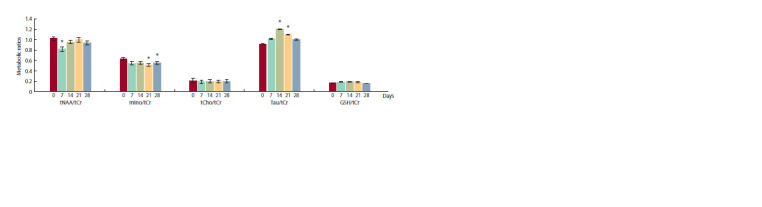
Dynamics of the relative levels of 5 metabolites (tNAA/tCr, mIno/tCr, Tau/tCr, GSH/tCr, tCho/tCr) with levels before (0 days) and 7, 14, 21, and 28 days after the intracranial injection. Here and in Fig. 3 and 4: * Significant difference from 0 days ( p < 0.05; Student’s t-test for dependent samples).

Evaluation of indicators of brain cell viability, including tNAA/tCr (indicator of neuronal viability), mIno/tCr (indicator of glial cell viability), tCho/tCr (integrity of cell membranes), Tau/tCr (osmolyte, neuromodulator, and trophic factor) and GSH/tCr (antioxidant) showed that all of the metabolites returned to their initial levels on day 28 after injection ( p > 0.12), except mIno (Fig. 2).

The levels of metabolites reflected the viability and integrity of cells decreased in the first week of the study and then recovered two weeks after the injection significantly for tNAA/tCr ( p = 0.0152) and slightly for tCho/tCr ( p = 0.062) whereas the mIno/tCr level was significantly lower than the original on the 21st and 28th day after injection ( p = 0.0059 and p = 0.0484, respectively). A different picture was observed for Tau/tCr and GSH/tCr, which reflect the energy processes that take place in cells (see Fig. 2). It was found that the relative values of these metabolites increased significantly for Tau/ tCr and slightly for GSH/tCr with maximum at 14 days after administration: p = 0.001 and p = 0.054, respectively. The levels of Tau/tCr and GSH/tCr returned to the original values 28 days after the injection (see Fig. 2).

**Fig. 3:**
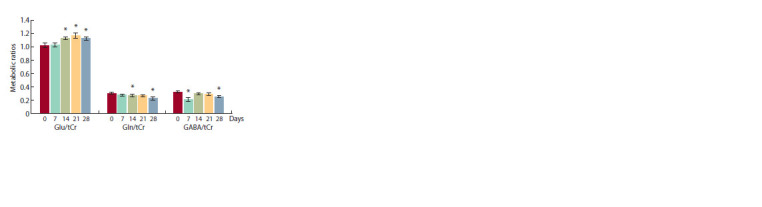
Dynamics of the relative levels of neurotransmitters (Glu/tCr, Gln/ tCr and GABA/tCr) before (0 days) and 7, 14, 21, 28 days after the intracranial injection.

The analysis of the relative level of metabolites performing the functions of neurotransmitters (Glu/tCr, Gln/tCr and GABA/tCr) also showed the dynamic processes in brain tissue after injection (Fig. 3). The Gln/tCr level progressively decreased from 7 to 28 days after the injection and was significantly lower than the original level on the 14th and 28th day after the surgery ( p = 0.028 and p = 0.026, respectively). The GABA/tCr level had the same dynamic profile, but on the 7th day, there was a dip before minimal values ( p = 0.015) with a following recovery and the next extinction on the 28th day ( p = 0.034). On the contrary, Glu/tCr increased, but only on 14 days after injection, maintaining high values up to 28 days ( p = 0.017, p = 0.019 and p = 0.045, respectively).

**Fig. 4.**
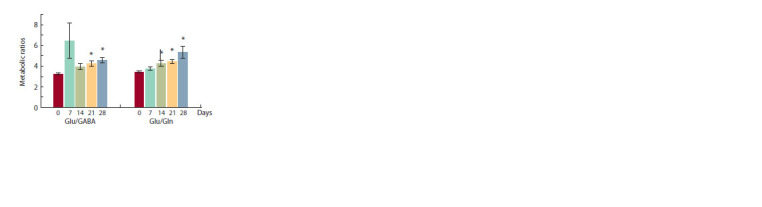
The Glu/GABA and Glu/Gln ratios before (0 days) and 7, 14, 21, 28 days after the intracranial injection

A particularly interesting results were found at analyzing the Glu/GABA and Glu/Gln ratios that reflect the effectiveness of neurotransmitters metabolism, because both the GABA and Gln have converted from Glu (Fig. 4). It was found that on the 7th day after the injection of the culture medium, the Glu/ GABA ratio significantly increased indicating a shift in the balance of neurotransmitters towards excitatory. These differences had disappeared by the 14th day post-injection, but with some progressive growth to 28th day. These differences were significant only for 21 and 28 days ( p = 0.024 and p = 0.002, respectively). Contrarily, the Glu/Gln ratio steadily increased during the experiment before a significant difference appeared on 14, 21 and 28 days after injection ( p = 0.003, p = 0.027 and p = 0.015, respectively).

## Discussion

The change in the tNAA/tCre ratio serves as an indicator of the dynamics of neuronal integrity and is an important biomarker of brain injury (Moffett et al., 2007). tNAA is involved in important processes such as lipid and myelin synthesis (Van Horn et al., 2017). In our study, the decrease in the tNAA level on day seven after surgery may indicate both potential neuronal loss and diffuse axonal injury characteristic of the early stages of brain trauma (Moffett et al., 2007), and its eventually recovery may indicate the stabilization process and the restoration of neurons and their viability

Another metabolite, mIno, is an osmolyte found mainly in astrocytes and glial cells of the microglia. Increased mIno levels are observed during both the subacute and chronic stages after experimental brain trauma, as well as after moderate or severe head trauma in humans (Brooks et al., 2001; Ashwal et al., 2004; Kierans et al., 2014). These changes are associated with reactive astrocytosis and microgliosis, accompanied by increased glial content and proliferation in response to brain injury (Ashwal et al., 2004), accelerated myelin breakdown, or hypertensive stress (Fisher et al., 2002). However, we found that mIno did not increase but rather decreased during the 28 days of the experiment, reaching the minimum values on day 21. Based on the data obtained, it can be assumed that the destruction of the integrity of the brain tissue caused by the needle injection of the culture medium did not lead to the changes in glial cell function

tCho, including choline compounds such as acetylcholine, phosphatidylcholine, and phosphocholine, is considered to be a product of nerve myelin disintegration and measures the membrane turnover (Xu et al., 2011; Li J. et al., 2017). In a study on rats (Lescot et al., 2010), two days after brain damage, a slight increase in the tCho level was detected, followed by recovery to the control value by seven days. In our study, we found a slight decrease in the tCho level seven days after the surgery, followed by its recovery to the baseline level, which is consistent with the behavior of the NAA level and may reflect the violation of membrane integrity and myelin degradation processes in the first week after surgery.

Another important indicator of brain cell activity is the balance of Tau levels. Tau is an endogenous amino acid synthesized in large quantities by neurons and astrocytes in the central nervous system. Tau acts as an osmolyte, neuromodulator, trophic factor, stabilizer of membrane integrity, and regulator of intracellular calcium homeostasis (Niu et al., 2018; Gupte et al., 2019). The significant increase in Tau at the injection region at 14 and 21 days post-injection, with the subsequent decrease at 28 days, may act as an adaptive brain tissue response to reduce the negative effects of the brain damage.

In response to the brain injury, markers of oxidative stress are produced in the brain, while levels of antioxidant defense enzymes (including GSH) are reduced (Rodríguez-Rodríguez et al., 2014). The addition of Tau to the culture medium in a neuron model with trauma led to an increase in GSH levels and, accordingly, a decrease in the effect of oxidative stress and in the levels of pro-inflammatory cytokines (Niu et al., 2018). In our study, it is likely that the increased Tau levels slowed the decline in GSH levels in the first three weeks after injection. The increase in the Tau level in the injection area led to a slight growth of the level of GSH in 14 days. Then there was a subsequent decrease in Tau levels by 21 and 28 days after the injection, ultimately accompanied by the reduction of GSH levels, which may indicate a completion of the traumatic process in 28 days after the traumatic event.

The Glu level in brain trauma models decreases starting from the first hours after trauma and remains at a low level up to two weeks post-exposure (Xu et al., 2011). This decrease may be due to an increased release of Glu into the synaptic cleft in response to brain injury, its capture by astrocytes, and its subsequent accelerated conversion to Gln, as evidenced by an increase in Gln (Guerriero et al., 2015; Van Horn et al., 2017). This is confirmed by the analysis of the Glu/Gln ratio. Changes in the Glu/Gln ratio may indicate neuronal death or glial cell abnormalities. In our study, the Glu/Gln ratio was the only indicator the change in which in the injected area intensified over time. There are two potential reasons for this. First, it may indicate a disturbance in the conversion of Glu to Gln in astrocytes, caused both by the transport of Glu into the astrocyte and its conversion to Gln under the action of glutamine synthetase. Second, the change in the Glu/Gln ratio may have been caused by hyperactivity of the reverse conversion of Gln to Glu in neurons, likely due to glutaminase hyperactivation (Guerriero et al., 2015; Van Horn et al., 2017). Regardless, both of these processes indicate a breakdown of the Gln to Glu conversion system specific to brain trauma processes

Analysis of the GABA level showed a two-wave decline: on 7 and 28 days. These results generally agree with those in the literature, where it was shown that in the acute phase of brain trauma, the GABA level decreases from the first day after injury (Harris et al., 2012). We attribute this decrease to the reduced conversion of Glu to GABA. The same effect can be caused by the accelerated uptake and conversion of GABA to Gln by oligodendrocytes (Van Horn et al., 2017). Additionally, the Glu/GABA ratio reflects the balance of excitatory and inhibitory neurotransmitters and its displacement may indicate the development of post-traumatic pathology, for example, to epileptogenesis (Cantu et al., 2015).

In a study on rats with mild traumatic brain injury caused by the blast, it was shown that the NAA level did not change, changes were observed only for mIno, Glu, and Tau, and only the Tau level was restored to the initial values after seven days, while the other metabolites showed an increase (Li Y. et al., 2021). In our study, metabolites characterizing the viability of neurons returned to their original level on day 28. However, the levels of mIno, as an indicator of the glial cells state, and the levels of neutotransmitters and their predictor (Glu, GABA and Gln) did not return to their initial values within 28 days, which may indicate some incomplete processes of tissue repair (Rodríguez-Rodríguez et al., 2014; Guerriero et al., 2015). All this results in longitudinal changes to the functional state of the brain cells obtained during the modeling surgery procedure of glioma.

Many studies have aimed to determine methods of early diagnosis of brain tumor diseases, including the use of in vivo 1H MRS (Porcari et al., 2016; Hyare et al., 2017). It is known that during the development of glioblastoma, especially at its later stages, the amount of NAA is significantly reduced. Also, at the early stages of tumor development, the level of mIno increases significantly, decreasing towards the later stage (Bulik et al., 2013). At the same time, our data show that the process of cell xenotransplantation itself leads to a decrease in NAA on the 7th day after the surgery and a decrease in the level of mIno on the 21st day. Besides, glioma cells that secrete Glu lead to an increase in extracellular Glu. Although Gln concentrations in the contralateral brain tissue in patients with glioblastoma were significantly elevated compared with the levels found in normal brain (Chaumeil et al., 2015). In our study, we found an elevated level of Glu and an increased level of Gln.

The data obtained on the dynamics of the level of neurotransmitters in the brain during the simulation of the xenotransplantation process, on the one hand, can be the result of surgical intervention, as well as the result of multiple MRS procedures using anesthesia. At the same time, there is evidence of minimal effects of isoflurane anesthesia on the metabolomic profile in animals (Menshanov, Akulov, 2015; Söbbeler et al., 2018).

Taking together, when using animal models of human glioma, researchers should clearly distinguish between the changes in neurometabolites that are a response to brain injury caused by the injection of cancer cells into the brain, and the processes that may indicate the early development of a brain tumor. Therefore, this is important for understanding how the level of metabolites changes during the process of tumor development.

## Conclusion

For modeling orthotopic xenotransplantation of glioma cells into the brain of mice, it is necessary to understand which metabolites are produced by a growing tumor and which are the result of surgery invasion of the tumor cells injection. The dynamic of nine neurometabolites in the mouse brain after needle injection with in vivo 1H magnetic resonance spectroscopy was studied. On the 28th day after injection, only metabolic levels of cells reflecting neurons’ viability in the area of the injection were restored. However, the levels of neutotransmitters and their predictor (Glu, GABA and Gln) did not return to their initial values within 28 days. So, the recovery processes are largely completed on the 28th day after the traumatic event in the brain tissue, leaving open the question of the neurotransmitter system impairment. It is important to keep in mind when modeling human glioblastoma in mice and monitoring new treatments. In addition, these results may be important at the development of approaches for non-invasive diagnostics of traumatic brain injury as well as recovery and rehabilitation processes of patients after certain brain surgeries.

## Conflict of interest

The authors declare no conflict of interest.
